# Bacterial Pneumonia in Horses: Insights from a 10-Year European Single-Centre Retrospective Study

**DOI:** 10.3390/vetsci13090901

**Published:** 2026-09-02

**Authors:** Sabita D. Stoeckle, Henrike Friedrich, Katharina C. Jensen, Heidrun Gehlen

**Affiliations:** 1 Surgery and Radiology, Equine Clinic, Freie Universität Berlin, Oertzenweg 19 b, 14163 Berlin, Germany; 2 Institute for Veterinary Epidemiology and Biostatistics, Freie Universität Berlin, Königsweg 67, 14163 Berlin, Germany

**Keywords:** equine bacterial pneumonia, equine abscessing pneumonia, equine bronchopneumonia, equine bacterial bronchitis, equine pleuropneumonia, predisposing events

## Abstract

Bacterial pneumonia in horses typically occurs secondary to predisposing factors that compromise defence mechanisms. This 10-year retrospective study evaluated bacterial pneumonia in 81 horses treated at the equine clinic of Freie Universität Berlin from January 2015 to December 2024. Horses infected with primary pathogens such as *Rhodococcus equi* or *Streptococcus equi* ssp. *equi* were not included in the study. The most common predisposing events were aspiration, mainly following oesophageal obstruction (37.0%), post-anaesthetic disease (27.2%), primary respiratory signs (24.7%), and other conditions (11.1%). Horses with aspiration were significantly older than the “other” groups. Tachypnoea, tachycardia, and fever were common but not universal. Bronchopneumonia was the most frequent diagnosis overall and predominated after aspiration, whereas pleuropneumonia was more common after general anaesthesia and in horses with primary respiratory signs. Bacteriological examinations were performed in 51 horses. Mixed infections were common, and *Streptococcus equi* ssp. *zooepidemicus* was frequently isolated. Resistant isolates were detected in most tested cases, and multidrug resistance was especially common in post-anaesthetic horses. Overall survival to discharge was 77.8% and did not differ significantly between predisposing events. However, initial lymphopenia, pleural effusion, and the need for thoracic drainage were associated with reduced survival. Anaerobic bacterial isolation, pulmonary abscessation, and diagnosis were associated with readmission because of respiratory signs. Most survivors had acceptable long-term outcomes, although recurrent respiratory signs were common.

## Simple Summary

Equine bacterial pneumonia typically occurs after predisposing events such as aspiration during oesophageal obstruction or general anaesthesia. This study examined 81 horses suffering from bacterial lung disease over a period of 10 years (January 2015–December 2024). Horses infected with primary pathogens such as *Rhodococcus equi* or *Streptococcus equi* ssp. *equi* were not included in the study. Most commonly, horses presented after aspiration (37.0%) and after general anaesthesia (27.2%). Fewer horses showed primary respiratory signs (24.7%) or had another history (11.1%). Horses with aspiration were significantly older than the other groups. Elevated heart and respiratory rate, as well as fever were commonly observed, but not present in all cases. Bronchopneumonia was the most frequent diagnosis and predominated after aspiration, whereas pleuropneumonia was more common after general anaesthesia and in horses with primary respiratory signs. Bacteriological examinations were performed in 51 horses. Mixed infections were common, and *Streptococcus equi* ssp. *zooepidemicus* was frequently isolated. Bacterial resistance was observed in most isolates, and multidrug resistance was especially common in post-anaesthetic horses. Overall survival to discharge was 77.8% and did not differ significantly between predisposing events. However, initial lymphopenia, pleural effusion, and the need for thoracic drainage were associated with reduced survival. Anaerobic bacterial isolation, pulmonary abscessation, and diagnosis were associated with readmission because of respiratory signs. Most survivors had acceptable long-term outcomes, although recurrent respiratory signs were common.

## 1. Introduction

Unlike in many other domestic species, clinically significant bacterial pneumonia in horses rarely develops as a primary disease but rather secondary to predisposing conditions such as viral respiratory infections, prolonged transportation, or aspiration following oesophageal obstruction [1,2,3,4,5,6]. In North America and Australia, transport-associated pleuropneumonia, commonly referred to as “shipping fever,” represents a well-documented condition, particularly in young Thoroughbred racehorses subjected to long-distance travel [2,3,4,5,6,7]. Head elevation, which also occurs during transport, promotes bacterial accumulation in the lower airways as well as proportion of neutrophils in tracheal washes [8]. In contrast, transport-associated respiratory disease is reported less frequently in European countries. This is possibly caused by shorter travel distances, although transportation remains an important risk factor for bacterial pneumonia [9].

Oesophageal obstruction, dysphagia of neurological origin, or unintentional mispositioning of a nasogastric tube may predispose horses to bacterial pneumonia by contaminating the lower airways with saliva, feed material, and oropharyngeal microorganisms [2,9,10,11,12].

Furthermore, general anaesthesia and surgical interventions have gained attention as potential contributors to postoperative respiratory disease in equine patients due to recumbency and anaesthetic drug-induced depression of respiratory function [2,9,13].

The microbial spectrum of equine bacterial pneumonia is typically dominated by opportunistic pathogens with *Streptococcus equi* ssp. *zooepidemicus* consistently reported as the most common isolate [2,4,9]. It frequently occurs alongside mixed aerobic and anaerobic bacterial populations, including *Staphylococcus* spp., *Pasteurella* spp., *Actinobacillus* spp., *Escherichia coli*, *Bacteroides* spp., and *Fusobacterium* spp. [2,7].

Despite extensive therapy including thoracic drainage and fibrinolytic therapy with recombinant tissue plasminogen activator (rTPA), the clinical outcomes remain variable [14,15]. Typically, the prognosis is negatively affected by factors such as extensive pleural effusion, fibrin accumulation, and azotaemia at presentation [15,16]. If multidrug-resistant bacteria are involved, therapeutic options are limited, highlighting the necessity of prudent antimicrobial use [17,18].

Comprehensive long-term retrospective studies investigating predisposing factors and outcomes of bacterial pneumonia in European equine populations remain limited. Predisposing events such as aspiration, transport, and general anaesthesia affect disease severity and outcome. This knowledge can aid the horse’s management and client communication.

Therefore, this study aimed to evaluate clinical cases of bacterial pneumonia over ten years at a major German equine referral hospital. Specifically, we investigated the relationship between predisposing events (aspiration, post-anaesthetic, primary or other) and clinical signs such as pleural effusion and/or abscess formation, microbiological findings as well as outcomes to identify factors associated with different clinical presentations. Horses infected with primary pathogens such as *Rhodococcus equi* or *Streptococcus equi* ssp. *equi* were not included in the study. Moreover, we aimed to reveal prognostic factors that influence discharge and readmission. Clinical signs, details, and anaesthesia, as well as the outcomes of the post-anaesthetic horses suffering from bacterial pneumonia included in this study, have been additionally described previously in a separate manuscript that is currently under review.

## 2. Material and Methods

### 2.1. Study Design and Case Selection

For this retrospective study, medical records of horses presented to the equine clinic of the Freie Universität Berlin between 1 January 2015 and 31 December 2024 were reviewed. Horses suffering from infectious lung disease were identified from keyword searches of the hospital management system (Vetera.net Campus 65.60.218, Vetera GmbH, Eltville am Rhein, Germany) using the terms “pneumonie”, “erguss”, “pleuritis”, and “drainage”, review of thoracic ultrasonography and radiography logs, and internal postoperative respiratory case lists.

Horses were eligible if they were ≥6 months old and had a diagnosis of bacterial pneumonia, including bronchitis, pneumonia, or pleuropneumonia. Inclusion required compatible clinical (e.g., coughing, increased respiratory rate), imaging, and laboratory findings, including fever ≥ 38.2 °C unless recently treated with non-steroidal anti-inflammatory drugs (NSAID), thoracic ultrasonographic and/or radiographic abnormalities, at least one abnormal inflammatory marker, and evidence supporting bacterial involvement. Evidence of bacterial infection included positive bacteriology, cytological evidence of bacteria or septic inflammation in tracheobronchial secretions or pleural fluid, including decreased glucose and increased lactate concentrations, or endoscopic evidence consistent with aspiration or contamination of the lower airways.

Horses were excluded if they were less than 6 months old, had non-infectious respiratory disease at presentation, traumatic pulmonary lesions, pulmonary oedema due to cardiovascular disease, viral or parasitic pneumonia, pulmonary neoplasia, or infection with primary pathogens such as *Rhodococcus equi* or *Streptococcus equi* ssp. *equi*. Predisposing events were grouped as primary infectious lung disease, aspiration, post-anaesthetic disease, or other conditions. Horses were classified as “primary infectious lung disease” if no predisposing factor was recognized. Patients were assigned to the group “aspiration” if they developed lung infection after oesophageal obstruction or if aspiration of feed material was visualized during endoscopic examination. Cases were categorised as post-anaesthetic if respiratory disease occurred within four weeks of anaesthesia. Horses that met the inclusion criteria but not any of the group criteria were assigned to the group “other”.

Of 295 horses initially identified, 81 met the final inclusion criteria. Of these, 21 horses did not fulfil all predefined criteria but were retained after individual case review because clinical, imaging, laboratory, bacteriological, or post-mortem findings supported bacterial respiratory disease. Cases with incomplete datasets were reviewed by both, a newly graduated veterinarian (H.F.) and a board-certified specialist in equine internal medicine (Diplomate ECEIM, S.D.S). For each individual case, clinical, diagnostic imaging, laboratory, bacteriological, or in case of death, post-mortem findings were reviewed. Based on these findings, it was decided whether a case was eligible for inclusion or not.

### 2.2. Data Collection

Collected information included signalment, history, vital parameters, laboratory data, diagnostic imaging, endoscopic findings, bacteriology, antimicrobial susceptibility, treatment, and outcome. Initial values were defined as those recorded at first presentation or, in hospitalised postoperative cases, at first onset of respiratory signs. Age, breed, sex, month and season of disease onset, and relevant historical events were recorded. Heart rate, respiratory rate and rectal temperature at initial examination were recorded. When multiple measurements were available on the same day, mean values were used. Tachycardia was defined as heart rate > 44 beats/min, tachypnoea as respiratory rate > 18 breaths/min and fever as rectal temperature > 38.2 °C.

Laboratory variables included haematology, serum biochemistry, serum amyloid A, fibrinogen, and, if available, arterial blood gas results. The first available value during the disease episode was used. Furthermore, details on diagnostic evaluation of pleural fluid (white blood cell count (WBC), protein concentration, lactate concentration, glucose concentration, cytology) and cytology of tracheobronchial secretions were considered. Cytological preparations were stained using a modified Wright–Giemsa stain and examined for cellularity, inflammatory cell population and intracellular or extracellular bacteria. Thoracic ultrasonographic findings included lesion localisation, pulmonary abscessation, pleural effusion, estimated effusion volume, and intrathoracic fibrin deposition. Radiographic findings included pulmonary pattern, pleural effusion as well as abscess formation. Endoscopic findings included dorsal displacement of the soft palate, recurrent laryngeal neuropathy, and mucosal abnormalities of the pharynx, larynx, or trachea, including necrosis, inflammation, or haemorrhage.

### 2.3. Diagnostic Classification

Disease categories were assigned using imaging findings. Bronchial radiographic patterns were classified bronchitis, interstitial or broncho-interstitial radiographic changes and/or interstitial ultrasonographic abnormalities as bronchopneumonia, focal pulmonary opacities or ultrasonographic abscesses as abscessing pneumonia, and pulmonary abnormalities combined with pleural effusion as pleuropneumonia.

### 2.4. Bacteriology and Antimicrobial Susceptibility Testing

Samples submitted for bacteriology included tracheobronchial secretions, pleural fluid, and abscess material. Tracheobronchial secretions were collected trans-endoscopically using a sterile catheter whose tip was sealed with sterile gel to avoid contamination during passage through the endoscope.

Isolates were classified as Gram-positive, Gram-negative, or obligate anaerobic bacteria. *Streptococcus equi* ssp. *zooepidemicus* was recorded separately. Antimicrobial resistance was evaluated by antimicrobial class. Multidrug resistance was defined as resistance to more than three antimicrobial classes.

### 2.5. Treatment and Outcome

Treatment variables included antimicrobial use, number and class of antimicrobials administered, NSAIDs, bronchodilators, mucolytics, and surgical interventions. Surgical treatment included thoracic drainage, abscess drainage, thoracoscopy, or thoracotomy. Only antimicrobials prescribed by the clinic were analysed.

Outcome data were obtained from clinical records and a telephone owner questionnaire. Recorded outcomes included survival to discharge, duration of hospitalisation, recurrence of respiratory signs after discharge, readmission to the hospital, clinical findings at discharge, and long-term respiratory performance. The questionnaire assessed recurrent coughing, nasal discharge, fever, tachypnoea, further treatment, current athletic use, change in respiratory performance, and overall health status. The time between the follow-up and hospital discharge varied according to the time that had passed between the pneumonia and the data collection period.

### 2.6. Statistical Analysis

Statistical analyses were performed using IBM SPSS Statistics, version 29.0.2.0 (20) MacOS (IBM Corp., Armonk, NY, USA). Associations between categorical variables were analysed using the Pearson chi-square test. If more than 25% of cells had expected counts below 5, Fisher’s exact test was used for 2 × 2 contingency tables, while the Fisher–Freeman–Halton exact test was applied to larger tables. Associations between continuous and categorical variables were assessed using the Mann–Whitney U test for comparisons between two independent groups and the Kruskal–Wallis test for comparisons involving more than two groups, as appropriate for non-normally distributed data. Odds ratios (ORs) were calculated to evaluate potential negative prognostic factors. Dependent variables included discharge status, readmission, post-treatment respiratory performance, and length of the initial hospital stay. Significance was set as usual at *p* < 0.05.

## 3. Results

### 3.1. Study Population

Eighty-one horses were eligible for analysis. The study population comprised 44 Warmbloods (54.3%), eight Thoroughbreds or Arabians (9.9%), 10 ponies (12.3%), and 19 horses of other breeds (23.5%). There were 37 mares (45.7%), 36 geldings (44.4%), and eight stallions (9.9%). The median age of the horses was 15 years (range 0.5–32 years). Anamnestic factors predisposing horses for bacterial lung infection were aspiration in 30/81 horses (37.0%), post-anaesthetic lung disease in 22/81 (27.2%), primary respiratory signs in 20/81 (24.7%), and other conditions in 9/81 (11.1%). This included horses suffering from a wound infection (2/9), horses suffering from colic without apparent misplacement of a nasogastric tube (5/9), and horses with prior viral respiratory infections (2/9). Aspiration was almost exclusively associated with oesophageal obstruction (29/30), there was one iatrogenic aspiration event. Horses in the group “other” had a history of colic, viral infection, wound infection, or thrombophlebitis. Age differed significantly between predisposing events (*p* < 0.001). Horses with aspiration were older (median 20.5 years, range 2–32) than post-anaesthetic horses (median 10 years, range 0.5–27) and horses in the group “other” (median 4 years, range 1–24).

Most cases were presented in winter (24/81; 29.6%) and autumn (23/81; 28.4%), followed by spring (20/81; 24.7%) and summer (14/81; 17.3%). No significant differences were identified between season and predisposing event.

### 3.2. Clinical and Laboratory Findings

Initial tachycardia was present in 48/79 horses (60.8%), tachypnoea in 59/78 (75.6%), and fever in 46/78 (59.0%). Fever was most common in post-anaesthetic horses (16/21; 76.2%) and least common in horses with aspiration (11/28; 39.3%). WBC abnormalities varied between groups. Leukopenia was most frequent in horses with aspiration (12/30; 40.0%), whereas leucocytosis was most frequent in horses with primary respiratory signs (10/20; 50.0%) and with patients assigned to the group “other” (5/9; 55.6%). Details on the clinical data are given in Table 1. There were no statistically significant differences between the groups.

Lymphocyte counts differed statistically significantly between predisposing events (*p* = 0.005), with lymphopenia most common in horses with aspiration (23/29; 79.3%). Serum amyloid A concentration was measured in 22 horses and was increased in all of these. Fibrinogen was measured in 20 horses and was increased in 16/20 cases. Details on the laboratory data are given in Table 2.

### 3.3. Imaging and Endoscopy

Radiography and/or sonographic localisation of the lung lesions was available in 49 horses. Bilateral pulmonary changes predominated in all groups and were present in all horses with aspiration for which localisation was available.

Thoracic or pulmonary abscessation differed significantly between groups (*p* = 0.009). Abscesses were most common in horses in the “other” group (4/9; 44.4%) and horses following general anaesthesia (6/21; 28.6%), and least common after aspiration (1/30; 3.3%). Pleural effusion was also associated with predisposing events (*p* = 0.015). Pleural effusion was identified in 10/21 post-anaesthetic horses (47.6%), 8/20 horses with primary respiratory signs (40.0%), 3/30 horses with aspiration (8.6%), and 2/9 horses in the “other” group (22.2%). Fibrin depositions in the pleural fluid were recorded in 7/22 horses with documented pleural effusion. Endoscopy was performed in 52 horses. Upper airway dysfunction was identified in 14/52 horses, including dorsal displacement of the soft palate in five and recurrent laryngeal neuropathy in nine horses. Mucosal abnormalities of the upper airways or trachea were present in 31/52 horses and were significantly associated with predisposing event (*p* < 0.001). These lesions were most common in horses with aspiration (18/20; 90.0%) and post-anaesthetic bacterial lung disease (9/13; 69.2%). Necrosis was most common post-anaesthetically (6/13; 46.2%; *p* = 0.006), whereas erythema or inflammation was most common after aspiration (17/20; 85.0%; *p* < 0.001).

### 3.4. Diagnosis

Final diagnoses were bronchopneumonia in 40/81 horses (49.4%), pleuropneumonia in 22/81 (27.2%), bacterial bronchitis in 10/81 (12.3%), and abscessing pneumonia in 9/81 (11.1%). Diagnosis differed significantly between predisposing events (*p* < 0.001). Bronchopneumonia predominated after aspiration (25/30; 83.3%), whereas pleuropneumonia was most common in post-anaesthetic horses (10/22; 45.5%) and horses with primary respiratory signs (7/20; 35.0%). 

### 3.5. Bacteriological Examinations and Susceptibility Testing

In 51/81 cases, samples were submitted for bacteriological examinations. No bacterial growth was detected in five horses (9.8%). A single isolate was detected in 12/51 horses, whereas mixed bacterial infection was identified in 34/51 (Table 3).

Gram-positive bacteria were isolated from 41/51 horses and Gram-negative bacteria from 36/51. Anaerobic bacteria were identified in 18/51 horses, most commonly in post-anaesthetic cases. Antimicrobial susceptibility testing was available for 35 horses. Resistant isolates were identified in 31/35 (88.6%). Resistance patterns differed significantly between predisposing events (*p* = 0.038). Multidrug-resistant isolates were most common in postoperative horses (8/15; 53.3%) and were identified in both aspiration cases with resistant isolates. *Streptococcus equi* ssp. *zooepidemicus* was isolated from 28/51 horses. There were significant differences between the predisposing events regarding detection of *Streptococcus equi* ssp. *zooepidemicus* (*p* = 0.005). In horses with primary bacterial lung disease, the pathogen was identified in 37.5% (6/16); in post-anaesthetic horses, in 65% (13/20); in horses with aspiration, 40% (4/10); and in horses with other history, 40% (2/5) of the cases.

### 3.6. Treatment

All horses were treated with antimicrobials, and initially broad-spectrum antimicrobials were used. If required, the antimicrobial agent was adapted to the antibiogram. Additionally, the horses received NSAIDs (most commonly flunixin-meglumine) and, if required, dipyrone as an antipyretic drug.

Antimicrobial use differed significantly between predisposing events (*p* = 0.002). Horses suffering from aspiration or post-anaesthetic bacterial lung disease were typically treated with more antimicrobial agents than the other subgroups possibly due to required changes according to antibiogram or change of administration route (intravenous vs. oral application). Sulfonamides were the most frequently used antimicrobial class (46/72; 63.9%), followed by beta-lactams (36/72; 50.0%), aminoglycosides (35/72; 48.6%), and metronidazole (33/72; 45.8%). In 17/72 patients (21%), antimicrobial therapy included tetracyclines. Rifampicin (1.2%, 1/72), macrolides (3.7%, 3/72), and fluoroquinolones (7.4%, 6/72) were used considerably less frequently. NSAID or antipyretic use also differed significantly between groups (*p* < 0.001). It was administered in all postoperative and aspiration cases but only in 66.7% (12/18) of horses in the primary signs group and 75% (6/8) of the “other” group.

Supportive medication in the form of bronchodilators and mucolytics was used in 77.8% (14/18) of horses with primary signs, 86.4% (19/22) of postoperative cases, 92.9% (26/28) of aspiration cases, and 62.5% (5/8) of cases in the “other” group.

If required, horses received thoracic drains. This was required significantly more often in post-anaesthetic horses compared to those with predisposing events (5/22, 22.7%; *p* = 0.016). In contrast, no drainage procedures were performed in horses with aspiration (0/28) or in the “other” group (0/8), while 21.1% (4/19) of horses with primary bacterial lung infection required thoracic drainage.

Surgical abscess incision was performed in 5.6% (1/18) of horses with primary bacterial lung infection and in 9.1% (2/22) of post-anaesthetic cases, whereas no such procedures were carried out in the aspiration or “other” groups.

Details on horse age, predisposing event, diagnosis and treatment are available in Supplementary Table S1.

### 3.7. Outcome

Sixty-three horses (77.8%) survived to discharge. Survival did not differ significantly between predisposing events. Survival to discharge was 15/20 (75.0%) for horses with primary respiratory signs, 18/22 (81.8%) for postoperative horses, 22/30 (73.3%) for horses with aspiration, and 8/9 (88.9%) for horses in the “other” group.

Median duration of hospitalisation among survivors was 10 days (range 1–73). Postoperative horses had a median stay of 12.5 days, compared with 8 days for horses with primary respiratory signs, 8 days for horses with aspiration, and 7.5 days for horses in the “other” group. At hospital discharge, 23/63 horses still showed tachypnoea. Follow-up thoracic imaging was performed in 36 horses before discharge, and residual abnormalities were present in 35/36 (97.2%).

Overall, 13 of 63 discharged horses (20.6%) were re-admitted to the hospital with respiratory signs. Re-admission was significantly more common in post-anaesthetic horses (7/18; 38.9%; *p* = 0.003). No horse in the aspiration group was re-admitted to the clinic because of respiratory signs.

Owner follow-up was available for 53/63 surviving horses. Recurrent respiratory signs after hospital discharge were reported in 29/52 horses, most commonly in horses with primary bacterial lung infection (10/12; 83.3%). Additional treatment after hospital discharge was required in 17/52 horses, including antimicrobial treatment in 3 horses and inhalation therapy in 10 horses. Reduced respiratory performance was reported in 10/53 horses. Overall health was rated as very good by 26/53 owners (49.1%).

### 3.8. Prognostic Factors

Initial lymphopenia was associated with non-survival (*p* = 0.026). Horses with lymphopenia were approximately five times more likely to die than horses with lymphocyte counts within the reference interval (OR 4.67, 95% CI 1.22–17.87; *p* = 0.026).

Pleural effusion was also associated with non-survival (*p* = 0.037). Horses with pleural effusion were approximately three times more likely to die than horses without effusion (OR 3.43, 95% CI 1.14–10.29; *p* = 0.028).

Furthermore, the necessity of thoracic drain placement was negatively associated with survival (*p* = 0.008). Horses requiring thoracic drains were approximately eight times more likely to die than horses not requiring drainage (OR 8.19, 95% CI 1.85–36.37; *p* = 0.006).

Anaerobic bacterial isolation was associated with re-admission to the hospital (*p* < 0.001; OR 13.3, 95% CI 2.77–64.09). Re-admission was also associated with diagnosis (*p* = 0.014) and pulmonary abscessation (*p* = 0.007; OR 7.54, 95% CI 1.81–31.52).

Hospitalisation time was longer in horses with thoracic drainage (*p* < 0.001), pleural effusion (*p* < 0.001), lymphopenia (*p* = 0.036), and in horses diagnosed with abscessing pneumonia or pleuropneumonia (*p* = 0.002).

## 4. Discussion

This 10-year retrospective study (2015–2024) provides a comprehensive overview of the pathogenesis, clinical presentation, and outcomes of 81 horses treated for bacterial infections of the lower respiratory tract in a German equine teaching hospital. Like previous studies, the results highlight the significant impact of predisposing events on the development of bacterial pneumonia and identify prognostic markers, such as initial lymphopenia, the presence of pleural effusion, and the use of thoracic drains.

The study population had a median age of 15 years, but distinct age patterns were observed based on the primary predisposing event. Horses that were categorized as “aspiration” were significantly older, with a median age of 20.5 years, compared to those developing infection post-anaesthetically (10 years) or primary bacterial lung disease (11.5 years) groups. This suggests that geriatric horses may be more vulnerable to secondary pneumonia following oesophageal obstruction or dysphagia, potentially due to an increased risk for oesophageal obstruction due to age-related changes in dentation hindering proper mastication [19,20,21].

The study identified aspiration and postoperative events as the most common precursors to bacterial pneumonia with bronchopneumonia predominating after aspiration (25/30; 83.3%) and pleuropneumonia after anaesthesia (10/22; 45.5%) and in horses with primary respiratory signs (7/20; 35.0%). However, a recent study originating from North America found oesophageal obstruction and transportation as the main contributing factors to equine bacterial pneumonia [2].

Regarding the physical examination, tachypnoea was the most frequent clinical sign, present in (75.6%) of the horses followed by tachycardia in 60.8% of the cases, while fever was observed only in roughly 59% at initial examination. Other studies also report unchanged vital parameters and the absence of fever in some cases and claim it as a reason for missing early signs of equine bacterial lower respiratory disease [2,16]. Furthermore, clinicians must note that the absence of fever and/or normal vital parameters does not necessarily rule out infection, as pre-treatment with, for example, NSAIDs such as flunixn-meglumin may ameliorate clinical signs such as pyrexia when given to endotoxic horses [22]. Additionally, these may mask fever and other signs when given prophylactically, for example, in horses following choke or after surgery.

A pivotal finding of this research is the identification of initial lymphopenia as a significant negative prognostic indicator. Hallowell and colleagues also reported lymphopenia as a negative prognostic factor recently [2]. While persistent lymphopenia is a recognized predictor of mortality in human sepsis [23], its application in equine pneumonia is relatively novel and may provide useful for the assessment of prognosis and client communication, especially since it has been identified in more than one retrospective study in different geographic areas [2].

The study also reinforces the value of Serum Amyloid A (SAA) as a sensitive marker for acute inflammation whose measurement should be incorporated into the management of critically ill horses, because it responds fast to inflammatory stimuli and therapeutic changes in equine bacterial pneumonia [24].

Again, thoracic ultrasonography has proven to be a useful tool for early detection of pleural effusion and focal lung changes as well as for monitoring response to treatment [25,26,27]. The presence of a pleural effusion was also a major negative prognostic factor, increasing mortality risk by approximately three times (OR 3.43, 95% CI 1.14–10.29). Thoracic drainage was reserved for severe cases in which large effusion volumes compromised lung function. Although its use was associated with an eight-fold increase in mortality risk (OR 8.19, 95% CI 1.85–36.37), this likely reflects the inherent severity of the disease in patients requiring such invasive measures rather than a failure of the procedure itself.

An association between the duration of hospitalisation and the presence or extent of pleural effusion has previously been reported [28]. Similarly, in the present study, horses with pleural effusion required approximately twice as long in the hospital as horses without pleural effusion (median, 15 vs. 7.5 days). This finding further highlights the importance of closely monitoring the response to treatment and adjusting therapy when necessary. Ultrasonography is particularly useful in this context, as it allows the repeated, non-invasive assessment of pleural effusion and facilitates the monitoring of changes over the course of treatment [25,26,27].

Microbiological examination confirmed that mixed infections (66.7%) are common and that *Streptococcus equi* ssp. *zooepidemicus* remains a typically isolated pathogen [2,4,15,16]. A critical concern is the high rate of antimicrobial resistance (AMR); 88.6% of antibiograms showed resistance to at least one drug, and 52% of pathogens were multidrug-resistant. This underlines the importance of “Antimicrobial Stewardship” and the routine use of culture-based therapy to ensure targeted treatment and prevent the spread of resistance [17,18].

Interestingly, while anaerobic bacteria did not significantly reduce survival as reported earlier [25,29], their presence was associated with a 13-fold higher risk of re-presentation (OR 13.3, 95% CI 2.77–64.09) for recurring respiratory signs.

Despite the challenges of treating bacterial equine pneumonia, the overall survival rate of 77.8% is encouraging. Long-term follow-up via owner questionnaires showed that 49.1% of survivors had “very good” health, and only 12.3% showed permanently reduced performance. This indicates that with early detection and aggressive management, most affected horses can return to their previous level of athletic function [28].

At last, the limitations of this retrospective study need to be addressed. Typically, not all data are available for every subject as it is the case in this study as well. Which may have influenced the statistical analysis. Additionally, not all horses received the same treatment, and furthermore, due to advances in veterinary medicine, treatment standards changed over the 10 examined years. However, this also reflects a more realistic view on equine bacterial lung infections which makes our findings valuable Furthermore, the study was conducted at a single institution within a specific geographic region, which may limit the generalizability of the findings.

## 5. Conclusions

Bacterial pneumonia is a serious condition which can be responsive to treatment in equine patients. Identifying lymphopenia, pleural effusion, and the use of thoracic drains as primary negative prognostic markers allows for more accurate triage and better client communication regarding costs and expectations.

## Figures and Tables

**Table 1:** Initial clinical parameters of the horses presented for bacterial pneumonia.

		Primary Bacterial Lung Infection		Post-Anaesthetic		Aspiration		Other	
			*n*		*n*		*n*		*n*
Heart rate (beats per minute)	Minimum	26	20/20	36	20/22	32	30/30	36	9/9
	Median	46		48		50		48	
	Maximum	64		88		80		84	
Respiratory rate (breaths per minute)	Minimum	12	19/20	12	21/22	12	29/30	12	9/9
	Median	26		20		24		18	
	Maximum	57		58		60		36	
Temperature (°C)	Minimum	37.4	20/20	37.5	21/22	34.4	28/30	37.3	9/9
	Median	38.4		39.4		38.05		38.2	
	Maximum	39.5		40.3		40.0		39.1	

**Table 2:** Initial laboratory parameters of the horses presented for bacterial pneumonia.

		Primary Bacterial Lung Infection		Post-Anaesthetic		Aspiration		Other	
			*n*		*n*		*n*		*n*
White blood cells (G/L)	Minimum	2.58	20/20	2.47	22/22	1.24	30/30	4.35	9/9
	Median	10.25		9.72		5.65		11.51	
	Maximum	20.02		18.08		13.75		23.23	
Neutrophils (G/L)	Minimum	1.07	20/20	1.60	21/22	0.28	29/30	1.48	9/9
	Median	8.68		8.81		4.00		7.79	
	Maximum	16.95		13.64		12.33		18.27	
Lymphocytes (G/L)	Minimum	0.30	20/20	0.60	21/22	0.20	29/30	0.45	9/9
	Median	1.68		1.45		0.99		2.80	
	Maximum	5.35		3.83		2.34		3.86	
Lactate (mmol/L)	Minimum	0.30	17/20	0.70	16/22	0.60	27/30	0.60	9/9
	Median	1.05		1.00		1.90		0.90	
	Maximum	5.70		4.60		18.00		1.90	
Glucose (mg/dL)	Minimum	63.00	20/20	70.00	20/22	88.00	27/30	74.00	9/9
	Median	110.50		116.50		132.50		118.00	
	Maximum	296.00		167.00		309.00		220.00	
SAA (ug/mL)	Minimum	7.80	6/20	209.60	12/22	265.60	3/30	433.00	1/9
	Median	338.55		344.34		319.60		433.00	
	Maximum	1296.10		893.60		326.00		433.00	
Fibrinogen (mg/dL)	Minimum	237.50	6/20	234.00	8/22	401.00	2/30	218.00	4/9
	Median	469.50		432.75		428.00		392.50	
	Maximum	561.00		535.00		455.00		465.00	

**Table 3:** Pathogen detection in the different predisposing events.

Clinical Group	Horses with Bacteriological Examination *n*	No Pathogen Detected *n* (%)	Single Pathogen *n* (%)	Mixed Infection (>1 Pathogen) *n* (%)
Primary bacterial lung infection	16	3 (18.8%)	3 (18.8%)	10 (62.5%)
Post-anaesthetic	20	1 (5.0%)	6 (30.0%)	13 (65.0%)
Aspiration	10	1 (10.0%)	2 (20.0%)	7 (70.0%)
Other	5	0 (0%)	1 (20.0%)	4 (80.0%)
Total	51	5 (9.8%)	12 (23.5%)	34 (66.7%)

## Data Availability

The data presented in this study are available on request from the corresponding author due to data protection.

## Supplementary Materials

The following supporting information can be downloaded at: https://www.mdpi.com/article/10.3390/vetsci13090901/s1, Table S1: Data on individual age, predisposing events, diagnoses, and treatment.
